# Exponentially fitted numerical method for solving singularly perturbed delay reaction-diffusion problem with nonlocal boundary condition

**DOI:** 10.1186/s13104-023-06347-6

**Published:** 2023-06-05

**Authors:** Getu M. Wondimu, Mesfin M. Woldaregay, Gemechis F. Duressa, Tekle G. Dinka

**Affiliations:** 1 Applied Mathematics Department, Adama Science and Technology University, Adama, Ethiopia; 2 Mathematics Department, Jimma University, Jimma, Ethiopia

**Keywords:** Singularly perturbed problem, Reaction-diffusion problem, Nonlocal boundary condition, Primary 65L11, 65L12, 65L20, 65L70

## Abstract

**Objectives:**

In this article, a singularly perturbed delay reaction-diffusion problem with nonlocal boundary conditions is considered. The exponential fitting factor is introduced to treat the solutions inside the boundary layer which occur due to perturbation parameter. The considered problem has interior layer at $s = 1$ and strong boundary layers at $s = 0$ and $s= 2$. We proposed an exponentially fitted finite difference method to solve the considered problem. The nonlocal boundary condition is treated using Composite Simpson’s $\frac{1}{3}$ rule.

**Result:**

The stability and uniform convergence analysis of the proposed approach are established. The error estimation of the developed method is shown to be second-order uniform convergent. Two test examples were carried out to validate the applicability of the developed numerical method. The numerical results reflect the theoretical estimations.

## Introduction

Many problems in science can be described by differential equations involving small parameter and delay [1–3]. Such mathematical problems can be extremely difficult to solve exactly and, in such cases, approximate solutions are required. Various scientific and engineering processes can be modeled as integral terms over the spatial domain that appear inside or outside of the boundary conditions [4, 5]. Such problems are said to be nonlocal problems. Differential equations having nonlocal problems become singularly perturbed while the highest derivative is multiplied by a small parameter. Many physical phenomena are formulated as nonlocal mathematical models. For example, thermodynamics [6], plasma physics [7], heat conduction [8, 9], underground water flow and populace dynamics [10] can be decreased to the nonlocal problems with integration conditions. Singularly perturbed delay differential equations (SPDDEs) with nonlocal boundary conditions are observed to be an exciting and important type of problem, which plays a vital role in modelling a wide range of realistic phenomena and also broadly implemented in fields like bio-sciences, control-theory [11], HIV infection models [12], populace dynamics [13] and signal transition [14], and so forth.

The well posedness of singularly perturbed differential equations (SPDEs) with nonlocal boundary conditions was proved in [15, 16]. SPDEs with integral boundary conditions are an essential class of problems and are studied by several authors. The authors in [17] presented a numerical method based on FDM for solving a class of third order singularly perturbed convection diffusion type equations with integral boundary condition on a Shishkin mesh. Cimen and Cakir [18] construct an exponentially fitted difference scheme on an equidistant mesh for solving singularly perturbed nonlocal differential-difference problem with delay. The authors in [19] developed a numerical method based on FDM for solving a class of systems of singularly perturbed convection diffusion type equations with integral boundary conditions on a Shishkin mesh. Raja and Tamislevan [20] considered a class of system of singularly perturbed reaction diffusion equations with integral boundary conditions and developed a numerical method based on a finite difference scheme on a Shishkin mesh. In [21], the authors advanced a finite difference scheme on a suitable piecewise Shishkin type mesh for solving SPDDEs of convection-diffusion kind with integral boundary condition (IBC).

The authors in [22] investigated a class of third order SPDDEs of the convection-diffusion kind with IBC. They devised a numerical method depends on FDM with Shishkin mesh. Sekar and Tamilselvan [23] looked at a class of SPDDEs of convection-diffusion type with IBC. A FDM with suitable piecewise Shishkin type mesh was developed to solve the problem. The authors in [24] presented a numerical method depends on a FDM on Shishkin mesh to solve the third-order SPDDEs of reaction-diffusion kind with IBC. The authors in [25] used an exponentially fitted numerical scheme to solve SPDDEs of convection-diffusion kind with nonlocal boundary conditions. Debela and Duressa [26] improved the order of accuracy for the method proposed in [25]. Kumar and Kumari [27] developed the method based on the idea of B-spline functions and efficient numerical method on a piecewise-uniform mesh was recommended to approximate the solutions of SPDDEs with IBC.

The standard numerical schemes used for solving a class of SPDEs are sometime not well posed and fail to provide exact solution for very small perturbation parameter $\varepsilon$. Consequently, it is efficient to propose appropriate numerical schemes which are uniformly convergent. To the best of our knowledge, the singularly perturbed delay reaction-diffusion problem with nonlocal boundary conditions has not previously been numerically handled using an exponentially fitted numerical technique. The main aim of this work is to develop $\varepsilon$-uniform convergent numerical method for SPDDEs of the reaction-diffusion problem with nonlocal boundary conditions.

This article is organized in the following manner. In section "Introduction", a brief introduction of the given problem is discussed. In section "Properties of continuous problem", properties of continuous problem are given. In section "Formulation of numerical scheme", formulation of numerical scheme is given. Uniform convergence analysis is studied in section "Uniform convergence analysis". In section "Numerical examples and discussion", numerical examples and discussion are given. In section "Conclusion", conclusion of the article is given.

In this work, we use the following notations: $\Omega =(0, 2)$, ${\bar{\Omega }} = [0,2]$, $\Omega _1 = (0,1)$, $\Omega _2 = (1,2)$, ${\bar{\Omega }}^{2N} = \{ 0, 1, 2, \cdots , 2N\}$, $\Omega _1 = \{1,2,3,\cdots , N-1\}$, $\Omega _2 = \{N+1,N+2,\cdots , 2N-1\}$. *C* denoted as a generic positive constant that are independent of parameter $\varepsilon$ and 2*N* mesh points. We assume that $\sqrt{\varepsilon } \le C N^{-1}$. We used the maximum norm defined by $\left| \left| w\right| \right| _\Omega := \sup \left| w(s)\right| , s \in \Omega$ to study the convergence of the numerical solution.

## Properties of continuous problem

Consider a class of SPDDEs with nonlocal boundary condition

1 $$\begin{aligned} {\left\{ \begin{array}{ll} {\mathcal {L}}w(s) = -\varepsilon w''(s)+a(s)w(s) + b(s)w(s-1) =f(s),~~ s\in (0,2),\\ w(s) =\phi (s),~~~~ s \in [-1,0],\\ {\mathcal {K}} w(2) = w(2)-\varepsilon \int _{0}^{2}g(s)w(s)ds =L. \end{array}\right. } \end{aligned}$$

where, $\varepsilon$ is a small positive number $(0<\varepsilon \ll 1)$. Assume that $a(s)\ge \alpha >0$, $b(s)\le \beta <0,$ $\alpha + \beta >0,$ *f*(*s*), and $\phi (s)$ are sufficiently smooth functions and *g*(*s*) is non negative monotone function and satisfy $\int _{0}^{2}g(s)ds < 1$. The above assumptions ensure that the problem (1) has a unique solution $w \in X = C^0(\Omega )\cap C^1(\Omega ) \cap C^2(\Omega _1 \cup \Omega _2)$. The problem (1) is equivalent to

2 $$\begin{aligned} {\mathcal {L}} w(s) = F(s), \end{aligned}$$

with boundary conditions

3 $$\begin{aligned} {\left\{ \begin{array}{ll} w(s) =\phi (s), s \in (-1,0),\\ w(1^-) = w(1^+), w'(1^-)=w'(1^+),\\ {\mathcal {K}}w(2) = w(2)-\varepsilon \int _{0}^{2}g(s)w(s)ds= L. \end{array}\right. } \end{aligned}$$

where

$$\begin{aligned} {\mathcal {L}}w(s)= & {} {\left\{ \begin{array}{ll} {\mathcal {L}}_1 w(s) = -\varepsilon w''(s)+a(s)w(s), ~~~~~~~s \in \Omega _1 = (0,1), \\ {\mathcal {L}}_2 w(s) = -\varepsilon w''(s)+a(s)w(s)+b(s)w(s-1),~~~ s \in \Omega _2 =(1,2). \end{array}\right. } \\ F(s)= & {} {\left\{ \begin{array}{ll} f(s)-b(s)\phi (s-1),~~~~ s\in \Omega _1, \\ f(s), ~~~~~~s \in \Omega _2. \end{array}\right. } \end{aligned}$$

### Lemma 1

(Maximum principle): Assume $\theta (s)$ be any function such that $\theta (0)\ge 0$, ${\mathcal {K}}\theta (2)\ge 0$, ${\mathcal {L}}_1\theta (s)\ge 0,~ \forall s \in \Omega _1$, ${\mathcal {L}}_2\theta (s)\ge 0,~ \forall s \in \Omega _2$, and $\theta '(1+)-\theta '(1-) =[\theta '](1)\le 0$, then $\theta (s) \ge 0$, $\forall s \in {\bar{\Omega }}$.

### *Proof*

We use proof by contradiction. Let us construct test function

4 $$\begin{aligned} r(s) = {\left\{ \begin{array}{ll} \frac{1}{8}+\frac{s}{2},~~ s \in [0,1],\\ \frac{3}{8}+\frac{s}{4},~~ s \in [1,2]. \end{array}\right. } \end{aligned}$$

Note that $r(s)>0, \forall s \in {\bar{\Omega }}$, ${\mathcal {L}}r(s)>0$, $\forall s \in \Omega _1 \cup \Omega _2$, $r(0)>0, {\mathcal {K}} r(2)>0$ and $[r'](1)<0$. Let $\mu = \max \left\{ \frac{-\theta (s)}{r(s)}\right\}$. Then, there exists $s_0\in {\bar{\Omega }}$ such that $\theta (s_0)+\mu r(s_0) =0$ and $\theta (s) +\mu r(s)\ge 0, \forall s \in {\bar{\Omega }}$. Therefore, the function $(\theta + \mu r)(s)$ attains its minimum at $s=s_0$.

Suppose the lemma doesn’t hold true, then $\mu >0$.

Case (i): $$s_0 =0;$$ $$0<(\theta +\mu r)(0)=\theta (0)+\mu r(0) =0.$$

Case (ii): $s_0 \in \Omega _1,$ $0<{\mathcal {L}}_1(\theta +\mu r)(s_0)= -\varepsilon (\theta +\mu r)''(s_0)+a(s_0)(\theta +\mu r)(s_0)\le 0$. Case (iii): $s_0 =1,$   $0\le [\theta +\mu r]'(1) = [\theta '](1)+\mu r'(1) <0.$

Case (iv): $$s_0 \in \Omega _2$$, $$0<{\mathcal {L}}_2(\theta + \mu r)(s_0)$$

$$\begin{aligned} = -\varepsilon (\theta +\mu r)''(s_0)+a(s_0)(\theta +\mu r)(s_0)+b(s_0)(\theta + \mu r)(s_0-1)\le 0.\end{aligned}$$

Case (v): $$s_0 =2$$, $$0\le {\mathcal {K}}(\theta + \mu r)(2)= (\theta +\mu r)-\varepsilon \int _{0}^{2}g(s)(\theta +\mu r)(s)ds\le 0.$$

Take note that in every case, we ended up with a contradiction. Hence $\mu >0$ is impossible. Therefore $\theta (s) \ge 0$, $\forall s \in {\bar{\Omega }}$. $\square$

Since the operator ${\mathcal {L}}$ satisfy the above maximum principle, the continuous solution *w*(*s*) of the (2)-(3) is unique if it exists.

### Lemma 2

(stability Result): The solution *w*(*s*) for the problems in (1) satisfies the bound

5 $$\begin{aligned} {\left| w(s) \right| } \le \max \left\{ \left| w(0)\right| , \left| {\mathcal {K}}w(2)\right| , {\left| \left| {\mathcal {L}} w\right| \right| } \right\} . \end{aligned}$$

### *Proof*

This Lemma can be proved using Lemma 1 and by constructing a barrier functions as $\psi ^\pm (s) = \max \left\{ \left| w(0)\right| , \left| {\mathcal {K}}w(2)\right| ,\left| \left| {\mathcal {L}} w\right| \right| \right\} r(s) \pm w(s),~ s \in {\bar{\Omega }}$, where *r*(*s*) is a test functions in (4). $\square$

### Lemma 3

Let $w \in C^2({\Omega })$ be the solution of (1). Then, for $k = 1, 2, 3, 4$,

6 $$\begin{aligned} {\left| \left| w^{(k)} \right| \right| } \le C\left( 1 + \varepsilon ^{-k/2} \right) . \end{aligned}$$

### *Proof*

Using Lemma 2 and applying arguments as given in [28] this lemma gets proved. $\square$

## Formulation of numerical scheme

The problems in (1) manifest strong boundary layers at $s=0$ and $s=2$ and have interior layer at $s=1$. Due to a dependence of *a*(*s*) and *b*(*s*) on spatial variable *s*, we cannot solve the problem analytically. With *N* identical mesh points, the range [0, 2] is separated into $\Omega _1 = (0, 1)$ and $\Omega _2 =(1, 2)$. After that, we get $s_i=i h, i=0, 1, 2, \cdots , 2N$. The differential equation is obtained if we take into account the interval $s \in (0,1)$ and the coefficients of (1) are assessed on the midpoint of each interval.

7 $$\begin{aligned} {\left\{ \begin{array}{ll} -\varepsilon w''(s)+a(s)w(s) =f(s)- b(s)\phi (s-1),~~ s \in \Omega _1 = (0,1),\\ {w(0)} =\phi (0),\\ {w(1) = \gamma ,} \end{array}\right. } \end{aligned}$$

where $\gamma$ is any arbitrary constant. Now, we present exponentially fitted operator finite difference method (FOFDM) on the discretized domain $\Omega _1 =[0,1]$. From (7) we have

8 $$\begin{aligned} -\varepsilon w''(s)+a(s)w(s) =F(s),~~ s \in \Omega _1 = (0,1), \end{aligned}$$

where $$F(s)=f(s)- b(s)\phi (s-1)$$.

We employ the theory used in the asymptotic technique to solve a singularly perturbed BVPs to find an approximation to the solution of (8). In our scenario, the domain is separated into three sub-domains, two boundary-layer subdomains near $s=0$ and $s=1$ and one regular subdomain, and the boundary layer problem is changed to a regular problem by proper transformations using stretching variables. We looked at the asymptotic expansion solution to the problem in (8) based on the theory of singular perturbations presented in [29].

9 $$\begin{aligned} w(s,\varepsilon ) = \sum _{i=0}^{N}\left[ {w_i(s)} + v_i(\tau )+u_i(\eta ) \right] \varepsilon ^i, \end{aligned}$$

where $\tau = \frac{s}{\sqrt{\varepsilon }},~~\eta = \frac{1-s}{\sqrt{\varepsilon }}$. Then, the zeroth order of (9) asymptotic expansion is given as

$$\begin{aligned} w(s) =w_0(s)+v_0(\tau )+u_0(\eta ), \end{aligned}$$

where $w_0(s) =\frac{F(s)}{a(s)}$ is a solution of a reduced problem (1), which does not satisfy the boundary conditions, $v_0 = Ae^{-\sqrt{\frac{a(0)}{\varepsilon }s}}$ is the left boundary layer correction and $w_0 = Be^{-\sqrt{\frac{a(1)}{\varepsilon }}(1-s)}$ is the right boundary layer correction. Therefore, the asymptotic solution of the zeros order of (7) become

10 $$\begin{aligned} w(s) = w_0(s)+Ae^{-\sqrt{\frac{a(0)}{\varepsilon }s}}+Be^{-\sqrt{\frac{a(1)}{\varepsilon }}(1-s)} +{\mathcal {O}}(\varepsilon ), \end{aligned}$$

where *A* and *B* are determined using the given boundary conditions. Now, we separate the range [0, 1] into *N* equal parts with uniform mesh length *h*. Let $0 =s_0, s_1, \cdots ,s_{N}=1$ be the mesh points. Then, we have $s_i=ih;~ i =0, 1, \cdots N$. We choose $N_1$ and $N_2$ such that $s_{N_1} = \sqrt{\varepsilon }$ and $s_{N_2} = 1-\sqrt{\varepsilon }$. Then, in the range $[0,\sqrt{\varepsilon }~]$ the boundary layer at $s=0$ and in the range $[1-\sqrt{\varepsilon },1]$, the boundary layer will be at $s=1$.

At $s =s_i$, the above differential equations (7) can be written as

11 $$\begin{aligned} {\left\{ \begin{array}{ll} -\varepsilon w''(s_i) +a(s_i)w(s_i) = F(s_i),\\ w(0) =\phi (0)\\ w(N) = {\gamma }. \end{array}\right. } \end{aligned}$$

For convenience, we take $a(s_i)=a_i, w(s_i) = w_i, F(s_i) =F_i$. Now, consider finite difference for $w_i'' =\dfrac{w_{i-1}-2w_i+w_{i+1}}{h^2}$, $i= 1,2,3,\cdots , N-1$ and by substituting in (11), we obtain

12 $$\begin{aligned} -\varepsilon \left( \dfrac{w_{i-1}-2w_i+w_{i+1}}{h^2}\right) +a_iw_i =F_i. \end{aligned}$$

### Case I: left boundary layer

The problem of the form in (7) has left boundary layer at interval $[0,\sqrt{\varepsilon }]$. Then, the zeroth order approximation of asymptotic solution is given as

13 $$\begin{aligned} w(s) = w_0(s) +Ae^{\sqrt{\frac{a(0)}{\varepsilon }}s}+{\mathcal {O}}(\varepsilon ), \end{aligned}$$

where $w_0(s)$ is the solution of the reduced problem and we choose *A* as a suitable constant. Using Taylor series approximation for $w_0(i+1)h$ and $w_0(i-1)h$ up to first order, we obtain

14 $$\begin{aligned} w(s_{i+1})= & {} w_0(ih)+Ae^{-\sqrt{a(0)}(i+1)\rho }, \end{aligned}$$

15 $$\begin{aligned} w(s_{i-1})= & {} w_0(ih)+Ae^{-\sqrt{a(0)}(i-1)\rho }, \end{aligned}$$

where $\rho =h/\sqrt{\varepsilon }$ and $h=1/N$. To handle the oscillation of the perturbation parameter, we multiply exponentially fitting factor $\sigma _1$ for the term with a perturbation parameter as,

16 $$\begin{aligned} -\varepsilon \sigma _1 w''(s)+a(s)w(s) =F(s), \end{aligned}$$

with boundary conditions $w(0)=\phi (0)$ and $w(1) =\gamma$.

If $W_i$ is a discrete solution for *w*(*s*) at grid point $s_i$, the numerical method for (16) is written in operator form as

$$\begin{aligned} {\mathcal {L}}^hW_i =F_i,~~~~i =1,2,3,\cdots ,N-1. \end{aligned}$$

with boundary conditions $W(0) =\phi (0)$, $W(N) = \gamma$, where

17 $$\begin{aligned} {\mathcal {L}}^hW_i =-\varepsilon \sigma _1\left( \frac{W_{i-1}-2W_i+W_{i+1}}{h^2}\right) +a_iW_i = F_i. \end{aligned}$$

From (17), we have

$$\begin{aligned} -\frac{\sigma _1}{\rho ^2} \left( W_{i-1}-2W_i+W_{i+1}\right) = F_i-a_iW_i. \end{aligned}$$

Now, by taking the limit as $h\rightarrow 0$ and using (13)–(15) and manipulate some calculations, the exponential fitting factor is obtained as

18 $$\begin{aligned} \sigma _1 =\frac{\rho ^2 a(0)}{4} \left( \csc h\left( \frac{\rho }{2}\sqrt{a(0)}\right) \right) ^2. \end{aligned}$$

This will be a fitting factor in the left boundary layer.

### Case II: right boundary layer

In the interval $[1-\sqrt{\varepsilon },1]$, the right boundary layer will be on the right side near to $s=1$. Now we introduce the exponential fitting factor as

19 $$\begin{aligned} -\varepsilon \sigma _2\left( \frac{W_{i-1}-2W_i+W_{i+1}}{h^2}\right) +a_iW_i = F_i,~~i=1,2,3,\cdots ,N-1. \end{aligned}$$

with boundary condition $W(0) =\phi (0)$ and $W(N) = \gamma$.

Now, to introduce the fitting factor $\sigma _2$ on the right hand side, we use the right boundary layer asymptotic solution with outer layer

20 $$\begin{aligned} w_i = w_0(s_i)+Be^{-\sqrt{\frac{a(1)}{\varepsilon }}(1-s_i)}, \end{aligned}$$

where $w_0(s_i)$ is the solution of the reduced problem and *B* is arbitrary constant determined by using boundary condition. Using the same fashion as the left boundary layer case, the exponentially fitting factor is obtained as

21 $$\begin{aligned} \sigma _2 = \frac{\rho ^2 a(0)}{4} \left( \csc h\left( \frac{\rho }{2}\sqrt{a(1)}\right) \right) ^2. \end{aligned}$$

The required discrete problem become given as

$$\begin{aligned} {\mathcal {L}}^hW_i =-\varepsilon \sigma _2\left( \frac{W_{i-1}-2W_i+W_{i+1}}{h^2}\right) +a_iW_i = F_i,~~~ i=1,2,3,\cdots , N-1 \end{aligned}$$

with boundary conditions $W(0) =\phi (0)$ and $W(N) =\gamma$.

An exponential fitting factor over $\Omega _2 =(1,2)$ is analogously calculated as a fitting factor in $\Omega _1 = (1,2)$. In general, one can take an artificial viscosity (fitting factor) for the given problem on ${\bar{\Omega }}_i^{2N}$ as

$$\begin{aligned}\sigma (\rho ) = \frac{\rho ^2 a(0)}{4} \left( \csc h\left( \frac{\rho }{2}\sqrt{\alpha }\right) \right) ^2.\end{aligned}$$

Suppose that ${\bar{\Omega }}^{2N}$ denote a separation of [0, 2] into 2*N* sub-intervals such that $0=s_0<s_1<s_2<s_3<\cdots < s_N=1$, and $1<s_{N+1}<s_{N+1}<\cdots < s_{2N}=2$ with $h_i =s_i - s_{i-1}$, $h = 2/2N =1/N,~~i=1,2,\cdots ,2N$.

Case I: Consider equation (1) on the domain $\Omega _1 =(0,1)$ which is given by
  $$\begin{aligned} -\varepsilon \sigma (\rho ) w''(s)+ a(s)w(s) =f(s)-\phi (s-1).\end{aligned}$$
  Hence, the required difference equation becomes
  22 $$\begin{aligned} -\frac{\varepsilon \sigma }{h^2} W_{i-1} +\left( \frac{2\varepsilon \sigma }{h^2}+a_i \right) W_i -\frac{\varepsilon \sigma }{h^2} W_{i+1} = f_i -b_i\phi (s_i-N), \end{aligned}$$
  for $i =1,2,3,\cdots ,N$. Equation (22) can be rewritten as
  $$\begin{aligned}{A_i}W_{i-1} + {B_i}W_i+{C_i}W_{i+1} =H_i,\end{aligned}$$
  where $$A_i=-\frac{\varepsilon \sigma }{h^2},~~B_i=\frac{2\varepsilon \sigma }{h^2}+a_i,~~C_i=-\frac{\varepsilon \sigma }{h^2},~~H_i=f_i -b_i\phi (s_i-N).$$
Case II: Consider equation (1) on the domain $\Omega _2$. Then, equation (1) also has left boundary layer near $s=1$ and right boundary layer at $s=2$. Then, by applying exponentially fitted finite difference scheme, we obtain $-\varepsilon \sigma (\rho ) \left( \frac{w_{i-1}-2w_i+w_{i+1}}{h^2}\right) { + }~ a_iw_i+b_iw_{i-N}+\tau =f_i$, which is rewritten as
  23 $$\begin{aligned} E_iW_{i-1}+F_iW_i+G_iW_{i+1} + {b_iW_{i-N}} = f_i \end{aligned}$$
  where $$E_i = -\frac{\varepsilon \sigma }{h^2},~~F_i = \frac{2\varepsilon \sigma }{h^2}+a_i,~~G_i = -\frac{\varepsilon \sigma }{h^2}.$$
Case III: For $i=2N$, we approximate $\int _{0}^{2}g(s)w(s)ds$ using the composite Simpson’s $\frac{1}{3}$ rule.

24 $$\begin{aligned}&\int _{0}^{2}g(s)w(s)ds=\nonumber \\&\frac{h}{3}\left( g(0)w(0)+g(2)w(2)+2\sum _{i=1}^{2N-1}g(s_{2i})w(s_{2i})+4 \sum _{i=1}^{2N}g(s_{2i-1})w(s_{2i-1})\right) =L. \end{aligned}$$

Since, $w(0)=\phi (0)$, from (3), this equation can be rewritten as

$$\begin{aligned} -\frac{4\varepsilon h}{3} \sum _{i=1}^{2N}g(s_{2i-1})W(s_{2i-1})-\frac{2\varepsilon h}{3}\sum _{i=1}^{2N-1}g(s_{2i})W(s_{2i})+\left( 1-\frac{\varepsilon h g(2)}{3}\right) W(s_{2N})=L \end{aligned}$$

As a result, the fundamental schemes for solving (1) on the entire domain ${\bar{\Omega }} = [0, 2]$ are the schemes given in (22)–(23) and (24), together with the local truncation error of $\tau$.

## Uniform convergence analysis

The discrete solution corresponding to equation (1) are given as follows:

25 $$\begin{aligned} {\mathcal {L}}_1W_i= & {} -\varepsilon D^+D^-W_i+a_iW_i=f_i -b_i\phi _{i-N}, i = 1,2,3,\cdots N-1, \end{aligned}$$

26 $$\begin{aligned} {\mathcal {L}}_2W_i= & {} -\varepsilon D^+D^-W_i+a_iW_i+b_iW_{i-N}= f_i,~i = N+1,N+2,N+3,\cdots 2N-1, \end{aligned}$$

subject to the boundary conditions:

$$\begin{aligned} {\left\{ \begin{array}{ll} W_i = \phi _i,~~~ i=-N,-N+1,\cdots ,0\\ {\mathcal {K}}^NW_{2N}=W_{2N}-\sum _{i=1}^{2N}\dfrac{g_{i-1}W_{i-1}+g_iW_i+g_{i+1}W_{i+1}}{3}h_i~~~~\text {and}\\ D^-W_N =D^+W_N. \end{array}\right. } \end{aligned}$$

### Lemma 4

(Discrete Maximum principle): Assume

$$\begin{aligned} \sum _{i=1}^{2N}\dfrac{g_{i-1}+g_i+g_{i+1}}{3}h_i= {\lambda } <1~ \end{aligned}$$

and $\theta (s_i)$ be any function such that $\theta (s_0)\ge 0$, ${\mathcal {K}}\theta (s_{2N})\ge 0$, ${\mathcal {L}}_1\theta (s_i)\ge 0,~ \forall s_i \in \Omega _1^{2N}$, ${\mathcal {L}}_2\theta (s_i)\ge 0,~ \forall s_i \in \Omega _2^{2N}$, and $D^+(\theta (s_N))-D^-(\theta (s_N))\le 0$, then $\theta (s_i) \ge 0$, $\forall s_i \in {\bar{\Omega }}^{2N}$.

### *Proof*

Define the test function

27 $$\begin{aligned} r(s_i) = {\left\{ \begin{array}{ll} \frac{1}{8}+\frac{s_i}{2},~~ s_i \in [0,1]\cap \Omega ^{2N},\\ \frac{3}{8}+\frac{s_i}{4},~~ s_i \in [1,2]\cap \Omega ^{2N}, \end{array}\right. } \end{aligned}$$

Note that $r(s_i)>0, \forall s_i \in {\bar{\Omega }}^{2N}$, ${\mathcal {L}}r(s_i)>0$, $\forall s_i \in \Omega _1^{2N} \cup \Omega _2^{2N}$, $r(s_0)>0$, ${\mathcal {K}} r(s_{2N})>0$ and $[r'](N)<0$. Let $\mu = \max \left\{ \frac{-\theta (s_i)}{r(s_i)}; s_i \in {\bar{\Omega }}^{2N}\right\}$. Then, there exists $s_0\in {\bar{\Omega }}$ such that $\theta (s_0)+\mu r(s_0) =0$ and $\theta (s_i) +\mu r(s_i)\ge 0, \forall s_i \in {\bar{\Omega }}$. Therefore, the function $(\theta + \mu r)(s_i)$ attains its minimum at $s=s_k$.

Suppose the lemma doesn’t hold true, then $\mu >0$.

Case (i): $$s_k =0;$$ $$0<(\theta +\mu r)(0)=\theta (0)+\mu r(0) =0.$$

Case (ii): $$s_k \in \Omega _1^{2N},$$ $$0<{\mathcal {L}}_1(\theta +\mu r)(s_k)= -\varepsilon (\theta +\mu r)''(s_k)+a(s_k)(\theta +\mu r)(s_k)\le 0.$$

Case (iii): $$s_k =s_N,$$ $$0\le D[\theta +\mu r]'(s_N) = [\theta '](s_N)+\mu r'(s_N) <0.$$

Case (iv): $$s_k \in \Omega _2$$, $$0<{\mathcal {L}}_2(\theta + \mu r)(s_k) =$$

$$\begin{aligned} -\varepsilon (\theta +\mu r)''(s_k)+a(s_k)(\theta +\mu r)(s_k)+b(s_k)(\theta + \mu r)(s_k-N)\le 0.\end{aligned}$$

Case (v): $$s_k =s_{2N},~ 0\le {\mathcal {K}}(\theta + \mu r)(s_{2N})=(\theta +\mu r)s_{2N}$$

$$\begin{aligned}- \sum _{i=1}^{2N}\dfrac{g_{i-1}(\theta + \mu r)s_{i-1}+g_i(\theta + \mu r)s_i+g_{i+1}(\theta + \mu r)s_{i+1}}{3}h_i\le 0.\end{aligned}$$

Take note that in every cases, we arrive at a contradiction. Therefore $\mu >0$ is impossible. Hence, $\theta (s_i) \ge 0$, $\forall s_i\in {\bar{\Omega }}^{2N}$. $\square$

Since the operators ${\mathcal {L}}_1$ and ${\mathcal {L}}_2$ satisfy the above maximum principle, the discrete solution $W_i$ of the (25)-(26) is unique if it exists.

### Lemma 5

Let $\psi (s_i)$ be any mesh function. Then for $0\le i \le 2N$ we have the following estimate.

28 $$\begin{aligned} \left| \psi (s_i) \right| \le \max \left\{ \left| \psi (s_0) \right| ,\left| {\mathcal {K}}\psi (s_{2N}) \right| ,\max _{i\in \Omega _1\cup \Omega _2}\left| {\mathcal {L}}^{2N}\psi (s_i) \right| \right\} \end{aligned}$$

### *Proof*

The proof is follows from Lemma 4 and by constructing a barrier functions

$$\begin{aligned}\theta ^\pm (s_i) = \max \left\{ \left| \psi (s_0) \right| ,\left| {\mathcal {K}}\psi (s_{2N}) \right| ,\max _{i\in \Omega _1\cup \Omega _2}\left| {\mathcal {L}}^{2N}\psi (s_i) \right| \right\} r_i\pm \psi (s_i),~~\forall s_i \in {\bar{\Omega }}^{2N}.\end{aligned}$$

$r_i$ is a test function given in (27). $\square$

### Theorem 1

Let $w(s_i )$ and $W(s_i)$ be the continuous solution of (1) and discrete solutions of (22)-(24) respectively. Then, for sufficiently large *N*, the following truncation error estimate holds:

29 $$\begin{aligned} \sup _{1\le i\le 2N} \left| W(s_i)-w(s_i) \right| \le CN^{-2} \end{aligned}$$

### *Proof*

Let us define a local truncation error as

$$\begin{aligned} \left| {\mathcal {L}}^{2N} \left( W(s_i)-w(s_i) \right) \right|=\, &{} \left| {\mathcal {L}}^{2N}W(s_i)- {\mathcal {L}}^{2N}w(s_i)\right| \\=\, &{} \left| -\varepsilon \sigma D^+D^-W(s_i)-\left( -\varepsilon \frac{d^2}{dx^2}w(s_i) \right) \right| \\\le \,&{} \left| \varepsilon \frac{d^2}{ds^2}w(s_i)-\varepsilon \sigma D^+D^-W(s_i)\right| \end{aligned}$$

where $\sigma (\rho ) = \frac{\rho ^2 a(0)}{4} \csc h^2\left( \frac{\rho }{2}\sqrt{\alpha }\right)$ and $\rho = N^{-1}/\sqrt{\varepsilon }$. From Taylor series expansion we get the bounds as

30 $$\begin{aligned}{} & {} \left| D^+D^-W(s_i)\right| \le C\left| \dfrac{d^2W(s_i)}{ds^2}\right| ,\nonumber \\{} & {} \quad \left| \left( \dfrac{d^2}{ds^2}- D^+D^-\right) W(s_i)\right| \le CN^{-2} \left| \dfrac{d^4 W(s_i)}{ds^4}\right| . \end{aligned}$$

Using the bounds for the differences of the derivatives in (30) and based on the result given in [30], we have

$$\begin{aligned} \left| {\mathcal {L}}^{2N} \left( W(s_i)-w(s_i) \right) \right|\le & {} C\left( \left| \varepsilon \sigma -\varepsilon \right| \left| W''(s_i)\right| +\varepsilon h^2 \left| W^{(4)}(s_i)\right| \right) \\\le & {} \varepsilon CN^{-2}\left| \frac{d^4W(s_i)}{ds^4}\right| \end{aligned}$$

Here, the target is to show the scheme is convergent independent of the number of mesh points. By using the bounds for the derivatives of the solution in Lemma (3), we obtain

$$\begin{aligned} {\mathcal {L}}^{2N} \left( W(s_i)-w(s_i) \right)\le & {} \varepsilon CN^{-2}\left( 1+\varepsilon ^{-2}\right) \\\le & {} \varepsilon CN^{-2}+\varepsilon ^{-1}CN^{-2}\\\le & {} CN^{-2}, ~~\text {since}~~ \varepsilon ^{-1} >\varepsilon . \end{aligned}$$

Hence by discrete maximum principle, we obtain

31 $$\begin{aligned} \left| W(s_i)-w(s_i)\right| \le C N^{-2}. \end{aligned}$$

At the point $s_i=s_{2N}$, we have

$$\begin{aligned}{} & {} {\mathcal {K}}^{2N}\left( W(s_{i})-w(s_{i}) \right) \\=\, & {} {\mathcal {K}}^{2N}W(s_{2N}) - {\mathcal {K}}^{2N}w(s_{i}),\\=\, & {} \phi _r - {\mathcal {K}}^{2N}W(s_{2N}),\\=\, & {} {\mathcal {K}}w(s_{i})- {\mathcal {K}}^{2N}W(s_{2N}), \\=\, & {} w(s_{2N})-\varepsilon \int _{0}^{2}g(s)w(s)ds - \left( w(s_{2N}) -\varepsilon \int _{s_0}^{s_{2N}}g(s)w(s)ds\right) , \\=\, & {} \varepsilon \int _{s_0}^{s_{2N}}g(s)w(s)ds -\varepsilon \sum _{i=\,1}^{2N}\dfrac{g_{i-1}w_{i-1}+4g_iw_i+g_{i+1}w_{i+1}}{3}h\\\le & {} C\varepsilon h^4\left( w^{(4)}(\xi _1)+w^{(4)}(\xi _2)+\cdots + w^{(4)}(\xi _{2N}) \right) \\\le & {} C\varepsilon {h^4}\left| \left| \dfrac{d^4w(\xi _i)}{dx^4}\right| \right| \\\le & {} C\varepsilon {h^4}(1+\varepsilon ^{-2})\\\le & {} C\varepsilon {h^4}+Ch^4\varepsilon ^{-1}\\\le & {} C{h^2}\\=\, & {} CN^{-2}. \end{aligned}$$

By using the bounds for derivative of the solution in Lemma 3 and applying discrete maximum principle, we obtain

32 $$\begin{aligned} \left| \left| W(s_i)-w(s_i) \right| \right| \le CN^{-2}. \end{aligned}$$

Thus, the results of (31) and (32) shows (29). Hence the proof is complete. $\square$

## Numerical examples and discussion

Since the exact solution of the given examples is not available, we use a double mesh technique to compute the maximum pointwise absolute error of the presented method.

### Example 1

$$\begin{aligned}{} & {} -\varepsilon \frac{d^2w(s)}{ds^2}+5w(s)-w(s-1) =e^{-s}\\{} & {} w(s) = 1,~~s\in [-1,0]\\{} & {} {\mathcal {K}}w(2) = w(2)-\varepsilon \int _{0}^{2}\frac{s}{3}w(s) ds=0. \end{aligned}$$

### Example 2

$$\begin{aligned}{} & {} -\varepsilon \frac{d^2w(s)}{ds^2}+5w(s)-sw(s-1) =1\\{} & {} w(s) = 1,~~s\in [-1,0]\\{} & {} {\mathcal {K}}w(2) = w(2)-\varepsilon \int _{0}^{2}\frac{1}{6}w(s) ds =0. \end{aligned}$$

We define the maximum pointwise absolute error as $E^N_\varepsilon = \max _i \left| W_i^N -W^{2N}_i \right|$. where *N* is a number of mesh points. Next, we compute the $\varepsilon$-uniform error estimate by using the formula $E^N =\max _\varepsilon \left( E^N \right)$. We compute the rate of convergence of the method by using the formula $R^N_\varepsilon = \log _2\left( \dfrac{E^N_\varepsilon }{E^{2N}_\varepsilon } \right)$. In the same manner we compute the $\varepsilon$-uniform rate of convergence by using the formula $R^N= \log _2\left( \dfrac{E^N}{E^{2N}} \right)$. The assumption $\sqrt{\varepsilon } \le CN^{-1}$ is made only for theoretical purpose. The numerical method works for all $\varepsilon$ for our examples.

**Table 1:** Maximum absolute error and rate of convergence of the scheme for Example 1

$\varepsilon \downarrow N\mapsto$	$2^{8}$	$2^{9}$	$2^{10}$	$2^{11}$	$2^{12}$
$2^{00}$	1.5590e-07	3.8974e-08	9.7458e-09	2.4338e-09	5.7258e-10
	2.0000	1.9997	2.0016	2.0877	
$2^{-02}$	5.8936e-07	1.4735e-07	3.6837e-08	9.2184e-09	2.2931e-09
	1.9999	2.0000	1.9986	2.0072	
$2^{-04}$	2.0742e-06	5.1865e-07	1.2967e-07	3.2418e-08	8.1199e-09
	1.9997	1.9999	2.0000	1.9973	
$2^{-06}$	8.2661e-06	2.0681e-06	5.1714e-07	1.2929e-07	3.2323e-08
	1.9989	1.9997	1.9999	2.0000	
$2^{-08}$	3.4244e-05	8.5876e-06	2.1486e-06	5.3725e-07	1.3432e-07
	1.9955	1.9989	1.9997	1.9999	
$2^{-10}$	1.3923e-04	3.5237e-05	8.8363e-06	2.2108e-06	5.5280e-07
	1.9823	1.9956	1.9989	1.9997	
$2^{-12}$	5.3954e-04	1.4155e-04	3.5824e-05	8.9834e-06	2.2476e-06
	1.9304	1.9823	1.9956	1.9989	
$2^{-14}$	1.8128e-03	5.4440e-04	1.4281e-04	3.6140e-05	9.0627e-06
	1.7355	1.9306	1.9824	1.9956	
$2^{-16}$	3.9145e-03	1.8217e-03	5.4691e-04	1.4346e-04	3.6304e-05
	1.1035	1.7359	1.9307	1.9824	
$E^N$	3.9145e-03	1.8217e-03	5.4691e-04	1.4346e-04	3.6304e-05
$R^N$	1.1035	1.7359	1.9307	1.9824

**Table 2:** Maximum absolute error and rate of convergence of the scheme for Example 2

$\varepsilon \downarrow N\mapsto$	$2^{8}$	$2^{9}$	$2^{10}$	$2^{11}$	$2^{12}$
$2^{00}$	1.0861e-07	2.7323e-08	6.8515e-09	1.7155e-09	4.2928e-10
	1.9910	1.9956	1.9978	1.9986	
$2^{-02}$	6.6233e-07	1.6559e-07	4.1397e-08	1.0372e-08	2.5643e-09
	1.9999	2.0000	1.9968	2.0161	
$2^{-04}$	2.5661e-06	6.4162e-07	1.6041e-07	4.0103e-08	1.0065e-08
	1.9998	2.0000	2.0000	1.9944	
$2^{-06}$	9.6604e-06	2.4168e-06	6.0430e-07	1.5108e-07	3.7770e-08
	1.9990	1.9998	2.0000	2.0000	
$2^{-08}$	3.7499e-05	9.4020e-06	2.3522e-06	5.8816e-07	1.4705e-07
	1.9958	1.9990	1.9997	1.9999	
$2^{-10}$	1.4619e-04	3.6986e-05	9.2740e-06	2.3202e-06	5.8017e-07
	1.9828	1.9957	1.9989	1.9997	
$2^{-12}$	5.5373e-04	1.4516e-04	3.6729e-05	9.2100e-06	2.3042e-06
	1.9315	1.9827	1.9956	1.9989	
$2^{-14}$	1.8396e-03	5.5161e-04	1.4464e-04	3.6601e-05	9.1780e-06
	1.7377	1.9312	1.9825	1.9956	
$2^{-16}$	3.9534e-03	1.8351e-03	5.5055e-04	1.4439e-04	3.6537e-05
	1.1072	1.7369	1.9309	1.9825	
$E^N$	3.9534e-03	1.8351e-03	5.5055e-04	1.4439e-04	3.6537e-05
$R^N$	1.1072	1.7369	1.9309	1.9825	

**Fig. 1:**
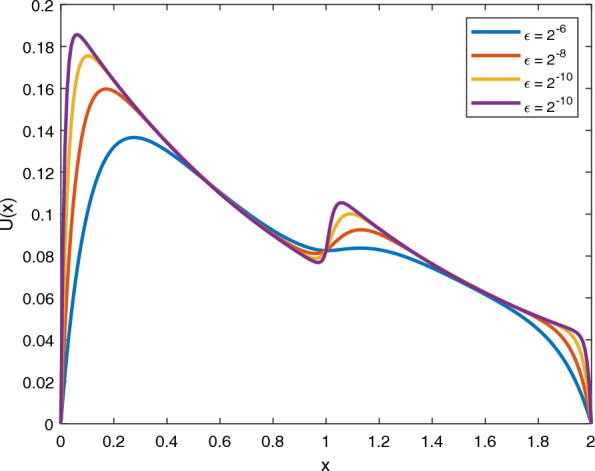
Graph of numerical solution which displays the existing layer for Example 1

**Fig. 2:**
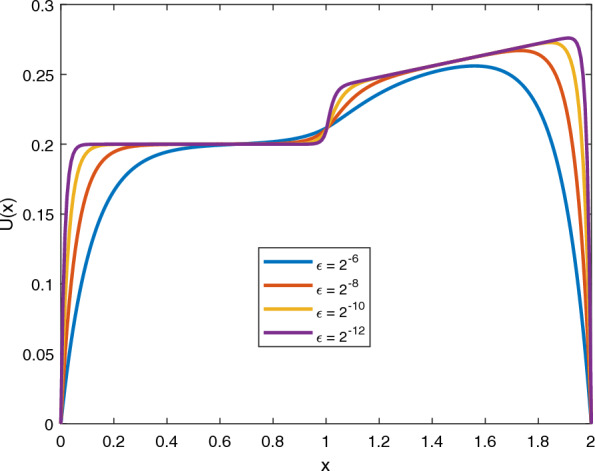
Graph of numerical solution which displays the existing layer for Example 2

The solutions of the given examples manifest strong boundary layer of thickness ${\mathcal {O}}(\sqrt{\varepsilon })$ close to $s=0$ and $s=2$ and interior layer at $s=1$. Tables 1 and 2 indicates the maximum absolute error and rate of convergence of the scheme for Example 1 and 2 respectively. The given tables suggested that the developed scheme is a parameter uniform convergent independent of mesh points with second-order of convergence. Figures 1 and 2 shows a graph of a numerical solution which displays the formation of boundary layer and interior layer as $\varepsilon$ goes to zero for Example 1 and 2 respectively.

## Conclusion

A class of singularly perturbed delay differential equations of reaction-diffusion problem with nonlocal boundary conditions is solved numerically. Due to the presence of a perturbation parameter on the higher order derivative the solution of the problem exhibit a boundary layers at $s =0$ and $s=2$ and interior layer at $s=1$. To obtain a numerical solution for this types of problems, we developed an exponentially fitted operator finite difference method (FOFDM) on a uniform mesh. The nonlocal boundary condition is approximated using Simpson’s $\frac{1}{3}$ rule. The stability and uniform convergence of the presented method are also investigated. Two test examples are considered for numerical experimentation to validate the applicability of the method. The developed numerical method is proved to be second-order uniformly convergent independent of perturbation parameter. Numerical experiments corroborate the theoretical findings. In future work, one can use a layer adapted mesh to solve the problem numerically.

## Limitations

The developed numerical scheme is not layer resolving method (i.e. there is no sufficient number of mesh points in the boundary layer region).

## Data Availability

No additional data is used for this study.

## Abbreviations

SPDEs: Singularly perturbed differential equations
SPDDEs: Singularly perturbed delay differential equations
IBC: Integral boundary conditions
FDM: Finite difference method
